# Bacterial community diversity of the deep-sea octocoral *Paramuricea placomus*

**DOI:** 10.7717/peerj.2529

**Published:** 2016-09-29

**Authors:** Christina A. Kellogg, Steve W. Ross, Sandra D. Brooke

**Affiliations:** 1 St. Petersburg Coastal and Marine Science Center, US Geological Survey, St. Petersburg, FL, United States of America; 2 Center for Marine Science, University of North Carolina at Wilmington, Wilmington, NC, United States of America; 3 Coastal and Marine Laboratory, Florida State University, St. Teresa, FL, United States of America

**Keywords:** Cold-water coral, Bacteria, Gorgonian, Submarine canyon, Microbiome

## Abstract

Compared to tropical corals, much less is known about deep-sea coral biology and ecology. Although the microbial communities of some deep-sea corals have been described, this is the first study to characterize the bacterial community associated with the deep-sea octocoral, *Paramuricea placomus*. Samples from five colonies of *P. placomus* were collected from Baltimore Canyon (379–382 m depth) in the Atlantic Ocean off the east coast of the United States of America. DNA was extracted from the coral samples and 16S rRNA gene amplicons were pyrosequenced using V4-V5 primers. Three samples sequenced deeply (>4,000 sequences each) and were further analyzed. The dominant microbial phylum was Proteobacteria, but other major phyla included Firmicutes and Planctomycetes. A conserved community of bacterial taxa held in common across the three *P. placomus* colonies was identified, comprising 68–90% of the total bacterial community depending on the coral individual. The bacterial community of *P. placomus* does not appear to include the genus *Endozoicomonas*, which has been found previously to be the dominant bacterial associate in several temperate and tropical gorgonians. Inferred functionality suggests the possibility of nitrogen cycling by the core bacterial community.

## Introduction

Cold-water corals provide critical three-dimensional habitat, creating biodiversity hot spots in the deep ocean (Buhl-Mortensen & Mortensen, 2005; Miller et al., 2012; Roberts et al., 2009). In addition to creating habitat for fishes and invertebrates, these corals are themselves landscapes for microbial associates (Penn et al., 2006; Yakimov et al., 2006). Studies on human gut microbes are revealing connections between internal microbiota and host-organism health and immunity (Turnbaugh et al., 2007); likewise, coral-associated microbiota are connected to the health and resilience of their hosts (Pantos et al., 2003; Ritchie, 2006). Bacterial pathogens or dysbiosis (microbial imbalance) have been linked to coral disease outbreaks on tropical reefs (e.g., Bythell, Pantos & Richardson, 2004; Mouchka, Hewson & Harvell, 2010) and mass die-offs of temperate corals (mainly gorgonians) (Bally & Garrabou, 2007; Hall-Spencer, Pike & Munn, 2007; Vezzulli et al., 2010). These studies have resulted in considerable attention and research into coral-associated microbes, particularly since the application of molecular tools and DNA sequencing in 2001 (Rohwer et al., 2001).

While much of the focus has remained on reef-building stony corals, increasing attention is being paid to gorgonians which also host diverse bacterial communities. Regardless of tropical, temperate, or deep-sea, most gorgonian bacterial microbiomes are dominated by Proteobacteria (Bayer et al., 2013; Brück et al., 2007; Correa et al., 2013; Duque-Alarcón, Santiago-Vázquez & Kerr, 2012; La Rivière, Garrabou & Bally, 2015; La Rivière et al., 2013; Penn et al., 2006; Ransome et al., 2014; Robertson et al., 2016; Sunagawa, Woodley & Medina, 2010; Vezzulli et al., 2013). However, Tenericutes (specifically *Mycoplasmas*) (Gray et al., 2011; Holm & Heidelberg, 2016) and recently Spirochaetes (Holm & Heidelberg, 2016; Lawler et al., 2016; Van de Water et al., 2016) have also been shown to be dominant or co-dominant in a few species. In gammaproteobacterial-dominated gorgonians, often the dominant genus was *Endozoicomonas* (Bayer et al., 2013; Correa et al., 2013; La Rivière et al., 2013; Ransome et al., 2014; Robertson et al., 2016; Vezzulli et al., 2013), while a common alphaproteobacterial genus was *Stenotrophomonas* (Bayer et al., 2013; Brück et al., 2007; Correa et al., 2013; Robertson et al., 2016). Comparable research on deep-sea corals has been slower, due to the high cost and difficulty of obtaining samples. Most of the focus has been on the deep-sea stony coral, *Lophelia pertusa* (Galkiewicz et al., 2011; Hansson et al., 2009; Kellogg, Lisle & Galkiewicz, 2009; Neulinger et al., 2008; Schöttner et al., 2009; Yakimov et al., 2006). However, microbial studies have been conducted on a few deep-sea octocorals (Gray et al., 2011; Lawler et al., 2016; Penn et al., 2006).

*Paramuricea placomus* (Linnaeus, 1758) is a plexaurid gorgonian coral endemic to the North Atlantic Ocean, occurring on both sides of the Atlantic basin (Buhl-Mortensen & Buhl-Mortensen, 2014; Simpson, Eckelbarger & Watling, 2005). In the western Atlantic, it has been documented on seamounts and deep reef areas along the east coast of the United States and into the Gulf of Mexico (Lumsden et al., 2007). Although commonly encountered as by-catch in fishing nets, little is known about *P. placomus* (Simpson, Eckelbarger & Watling, 2005). A temperate congener (*P. clavata*) in the Mediterranean was the focus of microbiological studies (La Rivière, Garrabou & Bally, 2010; La Rivière et al., 2013; Vezzulli et al., 2013; Vezzulli et al., 2010).

During a 2012 research expedition in and near Baltimore Canyon off the mid-Atlantic coast of the United States, we collected samples of *P. placomus* in order to describe the bacterial associates of a deep-sea *Paramuricea* species. Based on reports that have shown tropical (Littman et al., 2009; Rohwer et al., 2002), temperate (Van de Water et al., 2016), and cold-water (Kellogg, Lisle & Galkiewicz, 2009; Lawler et al., 2016) corals to have conserved bacterial communities associated with them, we hypothesized that *P. placomus* would also have a specific bacterial community with an identifiable core component shared by all individuals.

## Materials and Methods

### Sample site and collections

**Figure 1:**
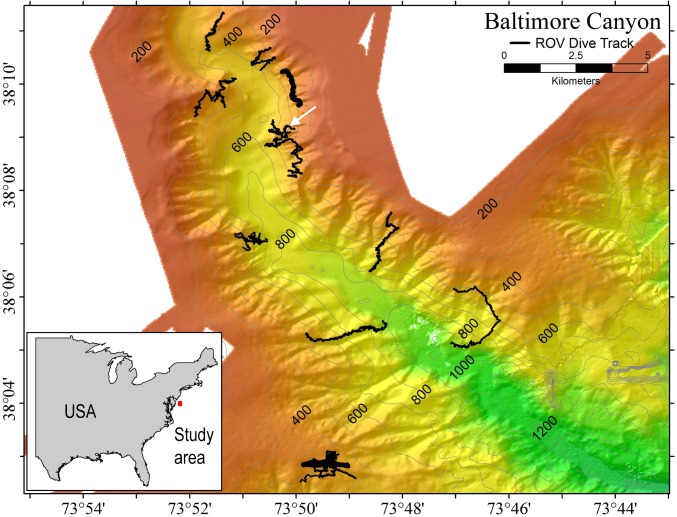
Multibeam sonar map of Baltimore Canyon showing *P. placomus* sample collection location (white arrow). Locations of all ROV dives made during the 2012 cruise are indicated by black lines. Depth contours are in meters.

**Figure 2:**
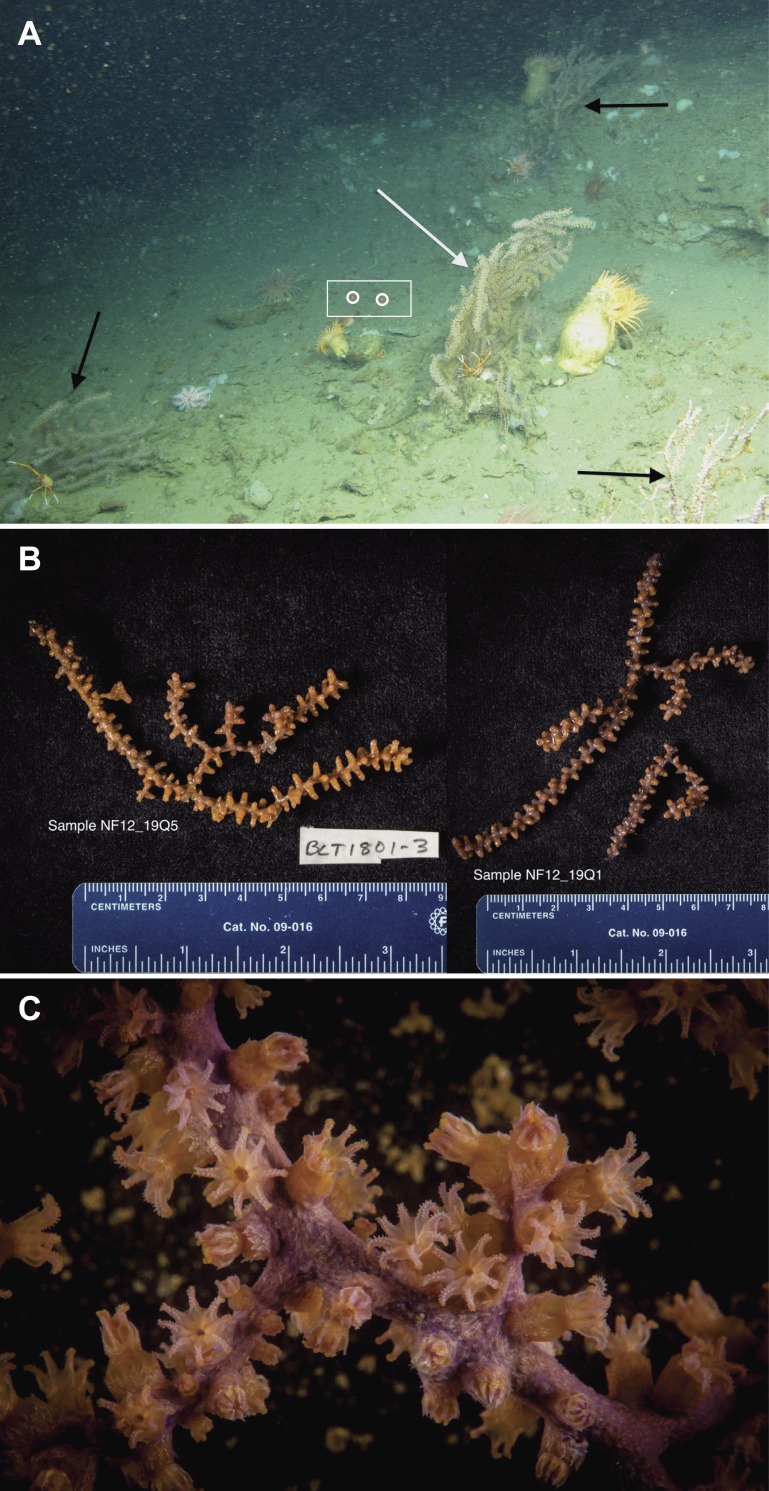
Images of *P. placomus*. (A) *In situ* photo of NF12.19Q1 (white arrow) showing general site rugosity and proximity (>1 m) of other *P. placomus* colonies (black arrows). For scale, red laser dots (circled in white and surrounded by a white box for clarity) in the center of the image are 10 cm apart; (B) Specimen photos of NF12.19Q5 and NF12.19Q1 showing differences in color pattern; (C) Close up image of *P. placomus* showing color variation between the stalk and the polyps. Image credit for (A): Deepwater Canyons 2012-Pathways to the Abyss, BOEM/NOAA-OER/USGS. Image credit for (B) and (C): Art Howard, Deepwater Canyons 2012–Pathways to the Abyss, BOEM/NOAA-OER/USGS.

Samples of *P. placomus* were collected from a single area in Baltimore Canyon (38°09.08′N, 73°50.26′W; white arrow, Fig. 1) on 12 September 2012, during a research cruise using the NOAA ship *Nancy Foster*. In spite of a number of other dives in this canyon (Fig. 1), this coral species was only observed in this one relatively small area. Collections were made using the remotely-operated vehicle (ROV) *Kraken II* (Univ. of Connecticut) during dive number ROV-2012-NF-19 between 10:30–11:30 Eastern Daylight Time (14:30–15:30 Coordinated Universal Time (UTC)). The sample site was a flat plateau with a depth range of 379–382 m, water temperature of 5.8–6.0 °C and salinity of 35.0. Samples NF12.19Q2, NF12.19Q5, NF12.19Q6 and NF12.19Q7 were all within one meter of each other and were collected without repositioning the ROV. This was done both to save time on a dive with multiple other objectives (repositioning the ROV can be time consuming and stirs up the substrate reducing visibility) and because the collections were being shared with a coral genetics group that was interested in the relationships of the close colonies. The ROV was moved a short distance away (2 to 3 m) to collect NF12.19Q1, at which point all the quivers dedicated to microbiology samples were filled. All coral colonies had adult galatheid squat lobster (*Eumunida picta*) associates, except NF12.19Q2, which hosted several galatheids that were too small to identify. Specimen NF12.19Q1 was a larger colony, with a dark purple stalk and mainly yellow polyps, in contrast to the other four specimens which had much paler, lavender stalks, and more variegated yellow and lavender polyps (Figs. 2B and 2C).

Small pieces of each coral colony (ca. 5–15 cm) were removed using the ROV’s manipulator arm and placed into individual polyvinyl chloride (PVC) quivers that had been washed, ethanol sterilized, filled with freshwater, and sealed with a rubber stopper while the ROV was on deck. The freshwater evacuated at depth when the quiver was opened to receive the coral sample, so that only seawater local to the coral samples was entrained during collection. Each coral sample was placed in a separate quiver and sealed at depth to prevent microbial contamination from other corals or different water masses during ascent. All collections occurred during 1 h. Upon recovery of the ROV (7 h after collection), the samples were removed from the quivers using ethanol-sterilized forceps, trimmed if necessary with ethanol-sterilized shears (to select a part of the coral sample that was not in contact with the ROV collection claw), and placed into individual, sterile 50 mL tubes. The tubes were filled with RNAlater solution (Life Technologies, Grand Island, NY, USA) to preserve the samples, placed at 4 °C overnight to allow the fixative to infiltrate the samples, and then transferred to −20 °C until ready for processing. If sufficient biomass remained, specimen photos were taken of the samples (Fig. 2B) and tissue was shared with a research group working on octocoral phylogenies.

### Nucleic acid extraction

Microbial community DNA was extracted from *P. placomus* following the protocol described in Sunagawa, Woodley & Medina (2010). Rather than grinding the sample, the protocol was modified by clipping a small piece (one polyp and its attached piece of central skeleton) from each octocoral sample using sterile forceps and shears and then placing it into a bead tube supplied with the PowerPlant DNA extraction kit (MO BIO, Carlsbad, CA, USA). Polyps were always collected from the part of the coral sample furthest from the end grasped during collection to limit any contamination from the ROV claw. The Sunagawa protocol was further modified by increasing the proteinase K (Ambion, Grand Island, NY, USA) incubation to 90 min from the protocol’s stated 60 min. Extracted DNA was quantified using the PicoGreen DNA quantification kit (Invitrogen, Grand Island, NY, USA) and the presence of bacterial DNA was confirmed by PCR amplification of 16S rRNA genes, using primers Eco8F (Edwards et al., 1989) and 1492R (Stackebrandt & Liesack, 1993), AmpliTaq Gold polymerase, and thermal cycled as follows: 1 cycle of 95 °C for 15 min; 30 cycles of 95 °C for 1 min, 54 °C for 1 min, and 72 °C for 2 min; and a final extension of 72 °C for 10 min.

### 16S rRNA gene pyrosequencing

The DNA extractions were sent to Selah Genomics (Greenville, SC) for sequencing. Each of the five *P. placomus* samples was amplified using barcoded primers (Integrated DNA Technologies, Inc., Coralville, IA, USA) targeting the V4-V5 hypervariable region of the 16S rRNA gene (563F/926R); V4-forward: 5′-AYTGGGYDTAAAGNG and V5-reverse: 5′-CCGTCAATTYYTTTRAGTTT (Claesson et al., 2010) Amplification was done using Roche’s FSHF (High Fidelity) polymerase and the following cycling parameters: 1 cycle of 95 °C for 2 min; 35 cycles of 95 °C for 30 s, 55 °C for 30 s, and 72 °C for 1 min; and a final extension of 72 °C for 4 min. The amplicons then underwent 454 pyrosequencing using Titanium FLX chemistry. Sequence data from all five samples were deposited in the NCBI Sequence Read Archive (SRA) under BioProject number PRJNA297333.

**Table 1:** Summary statistics for 454 sequencing of 16S rRNA genes from *P. placomus*.

Sample	Raw reads	Filtered reads	No. reads subsampled	Observed OTUs	Effective # of species	Shannon index	Chao1 richness	ACE richness	Simpson evenness
NF12.19Q1	4,393	4,300	4,300	105	54	3.98	107.80	108.20	0.0574
NF12.19Q2	7,577	7,268	4,300	107	36	3.58	117.91	115.17	0.0468
NF12.19Q5	6,197	5,635	4,300	168	97	4.57	170.36	173.47	0.0305
NF12.19Q6	404	262	–	–	–	–	–	–	–
NF12.19Q7	428	389	–	–	–	–	–	–	–

### Bioinformatics

**Figure 3:**
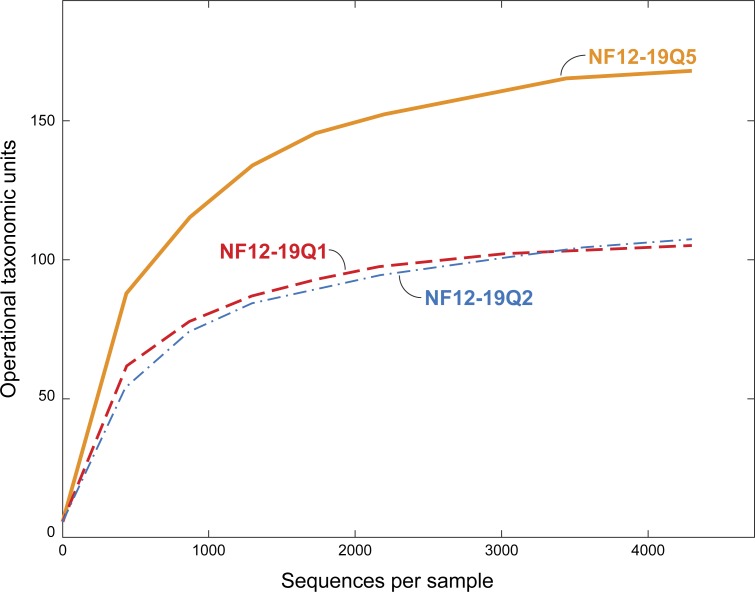
Rarefaction curves for bacterial diversity in three individuals of *P. placomus* collected from Baltimore Canyon. Although there were five samples collected, only three were successfully sequenced. Operational taxonomic units (calculated using a 97% sequence similarity cutoff) identified by 454 pyrosequencing of 16S rRNA genes.

A fully commented workflow describing the analysis conducted, as well as all the resulting output files, is available as a USGS data release at http://dx.doi.org/10.5066/F7HQ3WZZ (Kellogg, 2015). The software QIIME 1.9 (Caporaso et al., 2010b) was used to process and analyze the sequence data, and specific python scripts listed below were run within QIIME. The libraries were split and then quality checked using the following parameters: length between 200 and 700, quality score of 25 with a 50 bp running quality window, one primer mismatch allowed, and a maximum homopolymer run of 6 (Kunin et al., 2010). The data were denoised (Kunin et al., 2010; Quince et al., 2009) and then operational taxonomic units (OTUs) were picked using the pick_open_reference_otus.py script (Rideout et al., 2014) which combines a closed OTU picking against the reference database (Greengenes release 13_8; DeSantis et al., 2006) with a de novo method so as not to lose novel OTUs. We employed the usearch61 OTU-picking method (Edgar, 2010) in this script since it incorporates chimera-checking. Alignment was performed with PyNAST (version 1.2.2) (Caporaso et al., 2010a) and taxonomy was assigned with uclust (Edgar, 2010). Absolute singletons (OTUs that occur only once in the dataset) were removed from the OTU table as a default in this method. Any non-bacterial sequences (i.e., archaeal, eukaryotic, chloroplast, or mitochondrial) were removed in a post-OTU picking step. Two of the libraries sequenced poorly (Table 1), so the remaining three libraries were rarefied to 4,300 sequences and rarefaction curves were generated (Fig. 3). Diversity calculations were accomplished using alpha_diversity.py and beta_diversity.py. Effective number of species (the number of equally abundant species needed to obtain the same mean proportional species abundance as that observed in the data) was calculated by taking the inverse of the natural logarithm of the Shannon diversity value. Taxa relative abundance summaries were generated using summarize_taxa_through_plots.py, with the resulting data passed to Excel and then R-Studio (R Core Development Team, 2015) using the vegan (Oksanen et al., 2016) and ggplot2 (Wickham, 2009) packages to produce taxa summary plots. The core microbiome was analyzed with compute_core_microbiome.py with the minimum fraction of samples set at 100%. Comparisons of bacterial community dissimilarity between coral individuals were calculated using Similarity Percentages (SIMPER) from PRIMER (Clarke, 1993). SIMPER was run using abundance data with no transformation, using the sample names (NF12.19Q1, NF12.19Q2, NF12.19Q5) as factors.

## Results and Discussion

During this cruise 18 ROV dives were conducted from the head to the mouth of Baltimore Canyon, over sections of hardbottom (usually compacted mud) along both walls (Fig. 1). A depth range of 234–1,001 m was covered, with the bulk of the dives at 300–600 m. While other octocoral species, such as *Paragorgia arborea* and *Primnoa resedaeformis* were encountered at multiple locations in this canyon, *P. placomus* was only found at one location in Baltimore Canyon (Fig. 1). During a subsequent cruise in 2013, *P. placomus* was also sighted at a single location in Norfolk Canyon (37 02.96 N, 74 37.03 W; 448 m); unfortunately, the coral was identified from video review after the cruise, so no samples could be collected for comparison. In both cases, the *P. placomus* habitat was a flat rocky terrace on top of a steep wall. In Baltimore Canyon, the patch of *P. placomus* consisted of similarly-sized colonies (<1 m tall), with each colony typically less than one meter from another, possibly suggesting a single recruitment event (Fig. 2A). In Norfolk Canyon, the larger colonies were of similar size to those in Baltimore Canyon, but other smaller colonies were also observed. Like most corals, successful colonization of *Paramuricea* sp. requires hard substrata (Doughty, Quattrini & Cordes, 2013). Most of the octocoral species in these canyons were observed on underhangs or steep walls and boulders, presumably where sediment deposition was minimized. Colonies of *P. placomus*, however, were only found on relatively flat terrace-type habitat that had a veneer of sediment. Many flat terrace areas were observed in both canyons but distribution of this species was very limited, suggesting that other factors influence successful recruitment and colonization. These may include larval delivery (controlled by water currents), a lack of appropriate settlement cues, and sub-optimal water conditions (e.g., particle load) (Doughty, Quattrini & Cordes, 2013; Mienis et al., 2012). Also, sister species like *P. clavata* are surface brooders, which often results in short dispersal distances and was suggested as a reason for clumped distributions of the genus in the Gulf of Mexico (Doughty, Quattrini & Cordes, 2013).

### Bacterial diversity associated with *P. placomus* in Baltimore Canyon

**Figure 4:**
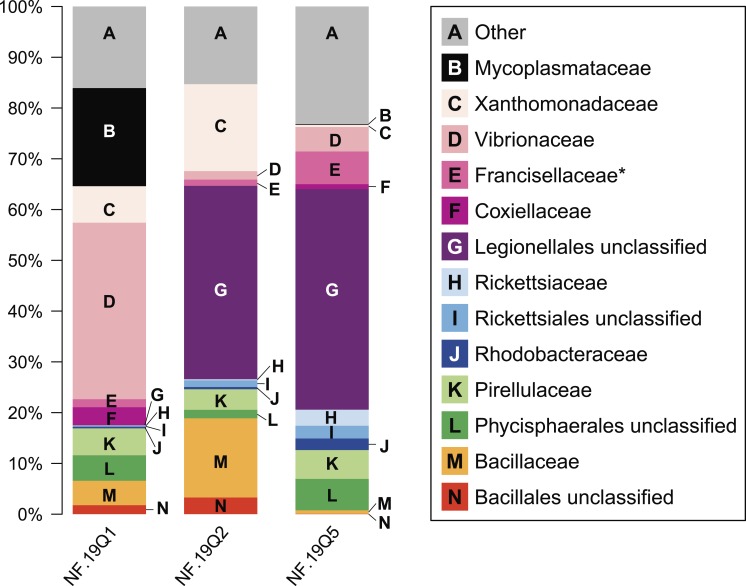
Relative abundance of common taxonomic groups in *P. placomus* samples. Families (or the nearest identifiable phylogenetic level) that represent ≥1% of the total taxa are shown. All remaining taxa are summarized as “Other” [A]. B = Tenericutes. Gammaproteobacterial groups are C–G, alphaproteobacterial groups are H–J, Planctomycetes are K–L, and Firmicutes are M–N. *See note in reference to Francisellaceae in Table 2.

All five of the Baltimore Canyon *Paramuricea* colonies share the same DNA sequence for the *mtMutS* gene (SC France & RW Clostio, pers. comm., 2015), which is consistent with their identification as *P. placomus* (Thoma, 2013). Because of the colonies close proximity to each other (<1 meter in most cases), they may not be genetically distinct. Due to the small number of successful sequence reads in two of the samples (NF12.19Q6 and NF12.19Q7), only sequences from the other three samples were further analyzed (Table 1, Figs. 3 and 4).

We chose to use an open OTU picking method so as to capture maximum diversity, including novel environmental sequences that do not have close matches in reference databases. The *P. placomus* Shannon index values (range = 3.58–4.57; Table 1) were similar to those seen for tropical stony corals like *Orbicella annularis* (Barott et al., 2011), but were higher than those seen for unimpacted *P. clavata* (range = 0.8–1.3) in the Mediterranean (Vezzulli et al., 2013), suggesting higher bacterial species richness compared to the temperate sister species (with the obvious caveat that the three studies did not use identical methodologies). Converting the Shannon index values to effective number of species (Hill, 1973), also known as true diversities (Table 1), allows a linear comparison of the magnitude of true diversity differences between two communities. The diversity of healthy *P. clavata* bacterial communities with Shannon index values of 0.8–1.3 (Vezzulli et al., 2013) is equivalent to that of communities with 2–4 equally-common species, compared to a range of 36–97 equally-common species (i.e., effective number of species) in *P. placomus* (Table 1). Using this metric, *P. placomus* bacterial communities are roughly 9 to 50 times more diverse than those associated with *P. clavata*.

As seen in most octocorals (Brück et al., 2007; Duque-Alarcón, Santiago-Vázquez & Kerr, 2012; Gray et al., 2011; Sunagawa, Woodley & Medina, 2010; Webster & Bourne, 2007), the bacterial community was dominated by proteobacterial sequences (>50% relative abundance). Other major phyla included the Firmicutes (10%) and Planctomycetes (10%). Tenericutes were 20% of sample NF12.19Q1, and less than 1% in the other two samples. Present at or below 1% relative abundance were Acidobacteria, Actinobacteria, Bacteroidetes, Chlamydiae, Chloroflexi, Cyanobacteria, Fusobacteria, Gemmatimonadetes, Lentisphaerae, and Verrucomicrobia.

At the microbial family level, pronounced differences occurred between sample NF12.19Q1 and samples NF12.19Q2 and NF12.19Q5; most notably, reductions in unclassified Legionellales and alphaproteobacterial groups, and increases in Vibrionaceae, and Mycoplasmataceae (Fig. 4). Similarity percentages (SIMPER) analysis (Clarke, 1993) showed the average dissimilarity between NF12.19Q1 and NF12.19Q2 or NF12.19Q5 was 50.63–51.03, and the groups responsible for more than 5% of that dissimilarity were Legionellales (11.9%), Vibrionaceae (7.1–9.4%), and Mycoplasmataceae (7.7–9.0%). The average dissimilarity between samples NF12.19Q2 and NF12.19Q5 was 44.04 and was driven by Xanthomonadaceae (7.5%) and Bacillaceae (6.8%).

*Mycoplasma* sp. sequences were previously found associated with other deep-sea corals, including the scleractinian *L. pertusa* (Kellogg, Lisle & Galkiewicz, 2009; Neulinger et al., 2009; Neulinger et al., 2008), and octocorals including a bamboo coral (Penn et al., 2006), *Cryogorgia koolsae* (Gray et al., 2011) and *Plumarella superba* (Gray et al., 2011). A phylogenetic comparison revealed two ‘coral’ *Mycoplasma* clades: the deep-sea octocoral clones clustered together with sequences from the tropical coral *Muricea elongata*, and the sequences from *L. pertusa* formed a separate cluster (Gray et al., 2011). We aligned the 329 bp *Mycoplasma* 16S rRNA sequence found in *P. placomus* against V4-V5 regions derived from nearly full-length 16S rRNA sequences available from *L. pertusa* (GenBank Accession number AM911412.1), bamboo coral (DQ395563.1), and *M. elongata* (DQ917875.1, DQ917898.1), to determine if it clustered in either of the two deep-sea coral clades. Note that we were unable to include other octocoral sequences because the clones were too short and did not include enough of the V4-V5 variable region for alignment. The *Mycoplasma* from *P. placomus* did not cluster with any of the other coral mycoplasmal sequences.

All three *P. placomus* samples showed higher relative abundance of gammaproteobacterial families (Fig. 4, letters C–G) compared to alphaproteobacterial families (Fig. 4, letters H–J). The bacterial community of temperate sister-species *P. clavata*, is heavily dominated by Gammaproteobacteria (>90%), whereas other corals from the family Plexauridae (*Cryogorgia koolsae* (Gray et al., 2011). *Eunicea fusca* (Duque-Alarcón, Santiago-Vázquez & Kerr, 2012) *Muricea elongata* (Ranzer, Restrepo & Kerr, 2006), and *Swiftia exertia* (Brück et al., 2007)) tend to have a roughly equal distribution of Alpha- and Gammaproteobacteria, but at much lower relative abundances (ca. 15–37% each).

The dominance of Gammaproteobacteria in *P. clavata* was due to the presence of a single bacterial genus, *Endozoicomonas*, as shown by both clone libraries (La Rivière et al., 2013)) and 16S rRNA amplicon pyrosequencing (Vezzulli et al., 2013). We did not detect this genus or its family (Hahellaceae) in our *P. placomus* samples. Rarefaction curves (Fig. 3) level off, indicating that the absence of *Endozoicomonas* in these samples is unlikely to be due to under sampling of the *P. placomus* bacterial communities. The genus *Endozoicomonas* dominated two other species of temperate gorgonians (Bayer et al., 2013; Ransome et al., 2014), and a recent study has suggested a coadaptation between clades of bacteria within Hahellaceae and several temperate gorgonian species (La Rivière, Garrabou & Bally, 2015). A small number of *Endozoicomonas* sequences were detected in the bacterial communities associated with the deep-sea coral *L. pertusa* (Kellogg, Lisle & Galkiewicz, 2009; Van Bleijswijk et al., 2015), and in two *Anthothela grandiflora* samples from Norfolk Canyon (Lawler et al., 2016). This would suggest that this bacterial group’s absence in *P. placomus* was not driven by a cold temperature or other depth-related limitation. Two studies of shallow-water scleractinian corals found that the corals located in their preferred habitats were dominated by *Endozoicomonas*, but had more diverse microbiomes in other habitats (Pantos et al., 2015; Roder et al., 2015). This raises questions of whether the absence of this bacterial group in *P. placomus* is driven by biogeography (due to the isolated nature of this particular coral population) or whether this could be a diagnostic feature of this species. In comparing *P. placomus* core taxa (Table 2) to the 16S rRNA amplicon pyrosequencing data from *P. clavata* (Vezzulli et al., 2013), the shared bacterial taxa for the coral genus *Paramuricea* are Actinobacteria, Rhodobacterales, Burkholderiales, and Vibrionales. Congeneric tropical acroporid corals harbored similar bacterial communities (Littman et al., 2009) and two deep-sea *Anthothela* species shared statistically indistinguishable bacterial communities (Lawler et al., 2016), so the lack of overlap between the bacterial communities of *P. placomus* and *P. clavata* was unexpected. However, perhaps it should not be surprising given (a) the differences in the studies’ methodologies and (b) the great differences between these corals’ environments: depth, pressure, temperature, and available food sources.

**Table 2:** Core bacterial taxa shared by three *P. placomus* samples and their relative abundance in each sample, as a percentage of the total taxa.

Core taxa					# OTUs	NF12.19Q1	NF12.19Q2	NF12.19Q5
Phylum	Class	Order	Family	Genus				
Actinobacteria	Actinobacteria	Actinomycetales	Microbacteriaceae	*Curtobacterium*	1	0.0	0.1	0.1
Actinobacteria	Actinobacteria	Actinomycetales	Propionibacteriaceae	*Propionibacterium*	1	0.3	0.6	0.9
Bacteroidetes	Flavobacteriia	Flavobacteriales	Flavobacteriaceae		1	1.0	0.0	0.2
Firmicutes	Bacilli	Bacillales			1	1.8	3.3	0.1
Firmicutes	Bacilli	Bacillales	Alicyclobacillaceae	*Alicyclobacillus*	1	0.4	0.1	0.0
Firmicutes	Bacilli	Bacillales	Bacillaceae	*Bacillus*	1	4.8	15.6	0.7
Planctomycetes	Phycisphaerae	Phycisphaerales			9	5.0	1.7	6.2
Planctomycetes	Planctomycetia	Pirellulales	Pirellulaceae		6	5.3	4.0	5.7
Proteobacteria	Alphaproteobacteria	Rhizobiales	Bradyrhizobiaceae	*Bradyrhizobium*	1	0.9	0.4	0.0
Proteobacteria	Alphaproteobacteria	Rhizobiales	Phyllobacteriaceae		1	0.6	0.1	0.0
Proteobacteria	Alphaproteobacteria	Rhodobacterales	Rhodobacteraceae		2	0.3	0.4	2.0
Proteobacteria	Alphaproteobacteria	Rhodospirillales	Rhodospirillaceae	*Magnetospirillum*	1	0.2	0.3	0.1
Proteobacteria	Betaproteobacteria	Burkholderiales	Alcaligenaceae		1	0.0	0.2	0.1
Proteobacteria	Betaproteobacteria	Burkholderiales	Comamonadaceae		2	0.3	1.3	0.7
Proteobacteria	Deltaproteobacteria	NB1-j	JTB38		1	0.7	0.2	0.9
Proteobacteria	Epsilonproteobacteria	Campylobacterales			1	0.3	0.6	0.8
Proteobacteria	Gammaproteobacteria	Alteromonadales			2	0.2	1.7	0.5
Proteobacteria	Gammaproteobacteria	Alteromonadales	OM60		1	0.3	0.0	0.2
Proteobacteria	Gammaproteobacteria	Enterobacteriales	Enterobacteriaceae		1	0.3	0.5	0.2
Proteobacteria	Gammaproteobacteria	Legionellales			2	0.1	38.1	43.5
Proteobacteria	Gammaproteobacteria	Pseudomonadales	Moraxellaceae	*Acinetobacter*	1	0.4	0.5	0.2
Proteobacteria	Gammaproteobacteria	Pseudomonadales	Pseudomonadaceae	*Pseudomonas*	2	1.3	0.0	0.7
Proteobacteria	Gammaproteobacteria	Thiotrichales	Francisellaceae^*^	*Caedibacter*	3	1.6	1.2	6.4
Proteobacteria	Gammaproteobacteria	Vibrionales	Vibrionaceae		1	34.3	0.7	1.3
Proteobacteria	Gammaproteobacteria	Vibrionales	Vibrionaceae	*Enterovibrio*	1	0.1	0.9	3.3
Proteobacteria	Gammaproteobacteria	Vibrionales	Vibrionaceae	*Photobacterium*	1	0.4	0.0	0.2
Proteobacteria	Gammaproteobacteria	Xanthomonadales	Xanthomonadaceae		1	0.3	0.1	0.2
Proteobacteria	Gammaproteobacteria	Xanthomonadales	Xanthomonadaceae	*Lysobacter*	1	6.9	17.0	0.1
**TOTALS**						**68.1**	**89.6**	**75.3**

**Notes.**

* *Caedibacter* are actually incertae sedis, being closely related to both *Legionella* and *Francisella*, but placed in Francisellaceae by Greengenes database.

A value of 0.0 means <0.1%.

### Composition of conserved core bacterial community

Sequences shared by all three coral samples were examined to identify core bacterial taxa (characterized to the lowest possible taxon, down to genus, Table 2). It is recognized that this population of *P. placomu*s is both isolated and likely highly clonal, and therefore may not reflect all the bacterial diversity present across the large geographic range of this coral species, or could contain regionally-specific taxa. While other studies have included bacterial groups present in 30–50% of the coral samples as part of the core (Ainsworth et al., 2015), given the small sample size of this study we have opted for the most conservative approach; requiring the OTU be present in 100% of the samples. For each sample, the relative abundance of each taxon is shown in relation to the total taxa (Table 2). For samples NF12.19Q5 and NF12.19Q2 the core taxa make up 75 to nearly 90% of the total community, suggesting a strongly species-specific bacterial community. The core taxa constitute 68% of sample NF12.19Q1, in spite of its visibly different appearance at the family level (Fig. 4). This can be contrasted against the microbiome of the temperate gorgonian *Eunicella cavolini*, where only 7 of 2067 OTUs (less than 1%) were shared across 9 samples (Bayer et al., 2013).

A member of the *P. placomus* core (Table 2), *Propionibacterium*, was recently identified as a conserved member of the core microbiome across a number of tropical and mesophotic corals (Ainsworth et al., 2015). The authors used fluorescently-labeled probes to localize the *Propionibacterium* to the corals’ endosymbiotic algae (zooxanthellae) and speculated these bacteria had a role in facilitating the success of the dinoflagellate-coral symbiosis, and/or meeting the coral host’s energy requirements. Interestingly, *Propionibacterium* spp. have been cultivated or detected by molecular techniques from both azooxanthellate (Lawler et al., 2016; Santiago-Vázquez et al., 2007) and zooxathellate corals (Bayer et al., 2013; De Castro et al., 2010; Nithyanand, Manju & Pandian, 2011). This raises an interesting question as to whether these conserved bacteria have multiple roles, or play a different role in azooxanthellate corals.

The phylum Firmicutes is present in the *P. placomus* core, has been identified as a core component conserved across multiple tropical coral holobionts (Ainsworth et al., 2015), and appears to be a minor component in most corals, although more are being detected by pyrosequencing than were previously by clone libraries (e.g., Ainsworth et al., 2015; Lawler et al., 2016; Sunagawa, Woodley & Medina, 2010; Van de Water et al., 2015). However, we may still be underestimating the contribution of Firmicutes to coral microbiota due to spore-formers’ relative resistance to DNA extraction (Filippidou et al., 2015). Culture-based work revealed that bacterial communities associated with several tropical stony corals and mesophotic azooxanthellate corals include a number of *Bacillus* species (Beleneva, Dautova & Zhukova, 2005; Brück et al., 2007; Nithyanand & Pandian, 2009). Culture-independent work, including microarrays (Kellogg et al., 2014; Kellogg et al., 2013) and pyrosequencing (Morrow et al., 2012), also revealed the Bacillaceae family to be a common associate of tropical corals. While abilities vary based on species, *Bacillus* spp. are known to produce siderophores to acquire iron and are assumed to have important roles in carbon and nitrogen cycling (Logan & De Vos, 2009).

The *P. placomus* core bacterial community includes Alpha-, Beta-, Delta-, Epsilon- and Gammaproteobacteria (Table 2). Legionellales sequences have not been commonly detected in association with corals. Single clones of Legionellales were obtained from the skeletons of Mediterranean corals *Cladocora caespitosa* and *Balanophyllia europaea* (Meron et al., 2012) and less than 10 clones similar to *Legionella feeleii* were associated with diseased colonies of temperate gorgonian *Eunicella verrucosa* (Ransome et al., 2014). A recent study of three deep-sea coral species within the family Anthothelidae detected this order as a minor component of their bacterial communities (Lawler et al., 2016). This bacterial order is defined as comprising facultative and obligate intracellular parasites, many of which are associated with free-living protozoa. It therefore remains to be determined if Legionellales are direct associates of the gorgonian, or infect amoebae that are part of the holobiont microbiome (Garrity, Bell & Lilburn, 2005; Rowbotham, 1986).

In addition to the dominant phylotypes of Legionellales, another major component of the core gammaproteobacterial taxa, particularly for sample NF12.19Q1, is the Vibrionaceae, including the genera *Enterovibrio* and *Photobacterium* (Table 2). Vibrionaceae are a common component of coral microbiota, having been found in healthy tropical (Bourne & Munn, 2005; Chimetto et al., 2008; Daniels et al., 2011; Lampert et al., 2006) and cold-water corals (Galkiewicz et al., 2011; Gray et al., 2011). While this bacterial group has been linked to a number of diseases in tropical (e.g., Arotsker et al., 2009; Ben-Haim & Rosenberg, 2002; Cervino et al., 2004) and temperate (Bally & Garrabou, 2007; Hall-Spencer, Pike & Munn, 2007) corals, pathogenicity seems to be driven by water temperatures greater than 20 °C.

While other members of the order Xanthomonadales have been found in association with corals (Brück et al., 2007; Cárdenas et al., 2012; Rohwer et al., 2002), *Lysobacter* was only known from freshwater and soil environments (Christensen, 2005). However, this genus’s ability to degrade chitin (Christensen, 2005) would be useful for a coral host that feeds on zooplankton. *Lysobacter* are named for their antimicrobial activity, active against not only bacteria, but also yeasts, filamentous fungi, and nematodes (Christensen, 2005), suggesting this genus has a potential role in protecting and maintaining the microbial balance of the coral.

A component of the core microbiome of *P. placomus* was identified as Francisellaceae (Table 2 and Fig. 4). However, when those sequences were run through RDP Classifier (Wang et al., 2007) they were identified as Thiotrichales incertae sedis, genus *Caedibacter*. Further evaluation using BLAST (Altschul et al., 1990) showed that the closest matches in GenBank included uncultured *Caedibacter* clones derived from the coral *Orbicella faveolata* (FJ425613, FJ425621). The genus *Caedibacter* consists of endosymbionts of *Paramecium* and has been shown to be polyphyletic (Beier et al., 2002), including both Alphaproteobacteria similar to Rickettsiales and Gammaproteobacteria similar to both *Legionella* and *Francisella* (as seen here). As with Legionellales, the presence of this bacterial group hints at the presence of eukaryotic members (i.e., protist hosts) in these coral microbiomes.

### Core: Nitrogen cycling?

**Figure 5:**
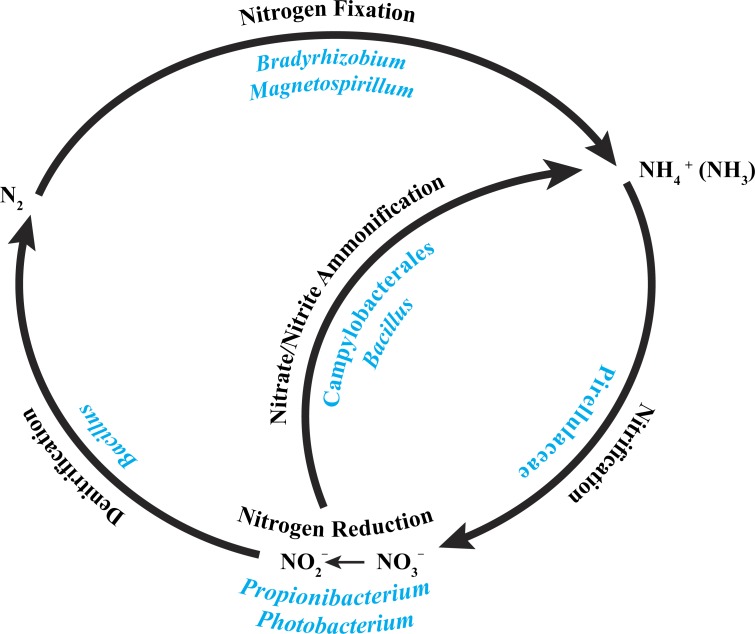
Core bacterial groups potential roles within the nitrogen cycle. A number of the bacterial groups present within the core microbiome of these three *P. placomus* samples were previously recognized for their roles within the nitrogen cycle. This diagram illustrates a simplified overview of the bacterial groups with their possible functions. This figure was adapted from one presented in Wegley et al. (2007).

A recent characterization of the core microbiome of the deep-sea octocoral *Anthothela grandiflora* revealed the possibility of a nearly complete nitrogen cycle, based on previously described abilities of particular bacteria present in the coral (Lawler et al., 2016). With this in mind, we examined the core bacterial community associated with the three *P. placomus* colonies (Table 2) and determined that a similar possibility exists for this coral species (Fig. 5).

The alphaproteobacterial genus *Bradyrhizobium* is typically an intracellular nitrogen-fixing symbiont in the root nodules of plants; however, some free-living strains of *Bradyrhizobium* have been observed to fix nitrogen with particular carbon sources and under low oxygen conditions (Kuykendall, 2005). Bacterial nitrogen-fixation has been documented in shallow-water corals (Cardini et al., 2015; Lesser et al., 2007; Shashar et al., 1994; Williams, Viner & Broughton, 1987), and *nifH* genes from *Bradyrhizobium* and other rhizobial species have been detected in three tropical coral species (Lema, Willis & Bourne, 2012). *Bradyrhizobium* spp. also have been identified previously in association with the deep-sea octocoral *Plumarella superba* (Gray et al., 2011), as well as in the tropical corals *Orbicella annularis* (Kellogg et al., 2013) and *Siderastrea siderea* (Kellogg et al., 2014). *Magnetospirillum* spp. are capable of fixing atmospheric nitrogen (Bazylinski et al., 2000) and can also use nitrate and ammonium as nitrogen sources (Schüler & Schleifer, 2005). Pirellulaceae are ammonia-oxidizing bacteria found in sponges (Gade et al., 2004; Mohamed et al., 2010) and the deep-sea octocoral *Alcyonium grandiflorum* (Lawler et al., 2016), and may be conducting nitrification. *Propionibacterium* (Allison & Macfarlane, 1989) and *Photobacterium* (Thyssen & Ollevier, 2005) have been shown to reduce nitrate to nitrite. *Bacillus* spp. have been shown to carry out denitrification (Verbaendert et al., 2011) and also nitrate/nitrite ammonification (Hoffmann et al., 1998). Lastly, members of the Campylobacterales are known to reduce nitrate and nitrite to ammonium (De Vries et al., 1980). Nitrogen cycling has recently been confirmed in the deep-sea coral *Lophelia pertusa* (Middelburg et al., 2015), and given that *P. placomus* is likely to have an inconsistent diet of nano-zooplankton and detrital particulates (Ribes, Coma & Gili, 1999), the ability to recycle and retain nitrogen would be beneficial (Fig. 5). Further work with metagenomics and transcriptomics remains to be conducted to confirm this hypothesis.

## Conclusions

Based on these three samples from the Baltimore Canyon population, the deep-sea plexaurid octocoral *P. placomus* has a species-specific bacterial community that shows very little overlap with previously characterized temperate gorgonians, including sister-species *P. clavata*. The bacterial community of this species is dominated by Proteobacteria but has similar diversity to that of tropical stony corals. Conserved core bacterial taxa comprise 68–90% of the total community, leaving roughly 10–30% individual variability between coral colonies. Additional sampling from other locations is required to confirm the consistency of these findings across the coral’s geographic range. The composition of the core suggests that nitrogen cycling may be carried out by the bacterial associates. This study is the first description of the bacterial microbiota of a deep-sea paramuricid species and provides a baseline for comparison by future studies to address questions regarding biogeography, ecology, and resilience of these corals to anthropogenic impacts and changing climate.

## Additional Information and Declarations

### DNA Deposition

The following information was supplied regarding the deposition of DNA sequences: NCBI Sequence Read Archive (SRA) under BioProject number PRJNA297333.

### Data Availability

The following information was supplied regarding data availability: Kellogg, 2015. Cold-water coral microbiomes (*Paramuricea placomus*) from Baltimore Canyon: raw and processed data. US Geological Survey data release: http://dx.doi.org/10.5066/F7HQ3WZZ.
